# A siramesine-loaded metal organic framework nanoplatform for overcoming multidrug resistance with efficient cancer cell targeting†

**DOI:** 10.1039/c9ra09923a

**Published:** 2020-02-14

**Authors:** Jiahui Liu, Menghuan Tang, Yanghao Zhou, Yijuan Long, Yuan Cheng, Huzhi Zheng

**Affiliations:** Key Laboratory of Luminescent and Real-Time Analytical Chemistry (Southwest University), Ministry of Education, College of Chemistry and Chemical Engineering, Southwest University Chongqing 400715 P. R. China zhenghz@swu.edu.cn; Department of Neurosurgery, The Second Affiliated Hospital of Chongqing Medical University Chongqing 400010 P. R. China

## Abstract

Cancer is the leading cause of death and the most important obstacle to increasing life expectancy. With the sophisticated design and research of anticancer drugs, multidrug resistance to chemotherapy has become more and more common. After the emergence of multidrug resistance, the development of a new drug is beset with difficulties. The repurposing of non-anticancer drugs is thus a timely strategy for cancer therapy. Here, we highlight the potential of repurposing siramesine, a central nervous system drug for antitumor research and we construct a metal organic framework-based nanoplatform for effective intracellular accumulation and pH-response siramesine release. The released drug induces lysosome membrane permeabilization, leading to lysosomal cathepsins leakage and then results in cell apoptosis. Due to the modification of folic acids, the constructed drug delivery nanosystem shows good biocompatibility and efficient cancer cell targeting. Importantly, the drug delivery system shows enhanced anticancer efficacy *in vitro*, which not only effectively kills cancer cells but also kills multidrug resistant cells. Thus, the drug delivery nanosystem constructed in this study is thought to become a promising anticancer agent for cancer therapy and even overcoming multidrug resistance, which provides good prospects for biomedical applications.

## Abstract 2

ZIF-8@Sira/FA induces the cancer cells apoptosis and then eliminates cancer cells from the inside through the lysosomal death pathway.

## Graphical abstract

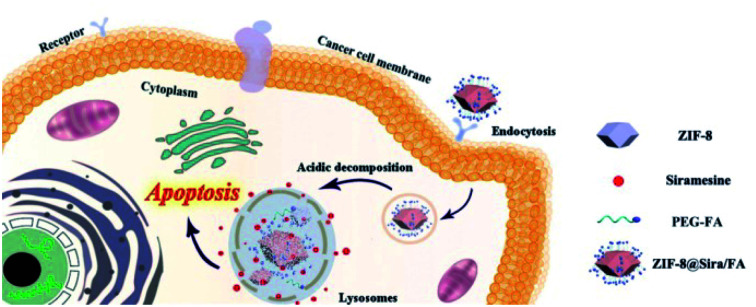

## 1. Introduction

Malignant tumors are still a huge threat to human health and well-being.^1^ Chemotherapy, as one of the most common methods for the treatment of cancer, has been extensively studied. However, the development of a new anticancer drug requires a lot of time and resources, for example, the costs of new cancer drug development increase rapidly and are currently in the range of $100 000 to $150 000 per year.^2^ Thus, the repurposing of Food and Drug Administration-approved drugs for treating diseases different from the ones they were originally approved for is a timely strategy, which is worthy of attention for the oncology community.^2^ These drugs can be tested immediately, and success rates are often higher than for new drugs in development, probably because their safety record is well-known. As the research goes on, some inexpensive drugs exhibit anticancer activity, such as disulfiram,^3^ metformin,^4^ and aspirin,^5^ which may be used as a promising strategy in tumor resistance. Siramesine (Sira) is the commercially available drug used in central nervous system with pharmacological activities like anticonvulsant, antidepressant, and sedative–hypnotic activities. But it was also found that siramesine had tumor toxicity and carried an excellent safety profile.^6^ Due to its wide range of applications, clear pharmacological effects and minor side effects, siramesine is safe and convenient for anticancer research. Therefore, the new use of old drugs based on siramesine has certain inspiration for the treatment of cancer.

For a long time, lysosomes were regarded as the cell's recycling bin, because they were involved in multiple cellular processes such as membrane repair, pathogen resistance and autophagy.^7,8^ Later, the concept of lysosome cell death was presented and the role of lysosomal cathepsin leakage as a significant mediator of cell decease was proposed.^6^ This triggers great interest in studying lysosomotropic detergents to induce lysosome membrane permeabilization (LMP) leading to cell death. Siramesine, as a lysosomotropic detergent, was confirmed to inhibit acid sphingomyelinase and trigger lysosomal cell death, which can kill cancer cells from the inside.^6^ Compared with traditional DNA damage drugs such as doxorubicin^9^ and camptothecin,^10^ siramesine can reduce the damage of chemotherapy to normal tissue, which has some enlightenment for the development of cancer treatment based on the combination of lysosomal reagent and nanomaterials.

The non-targeting free drugs have strong cytotoxic effects on normal body tissue and the excessive use of these small molecule drugs can easily lead to multidrug resistance (MDR), invasion and metastasis of tumors, which limits their clinical application.^11–13^ To overcome these drawbacks of free drugs, various types of drug nanocarriers have been exploited including liposomes, polymer nanoparticles and inorganic nanoparticles.^14–16^ These nanoscale drug delivery systems were reported to improve the efficacy of drugs by enhancing bio-distribution and preventing drug degradation as the result of the enhanced permeability and retention effect.^15,17^ Recently, metal organic frameworks (MOFs) for efficient drug delivery have attracted a lot of attention owing to their simple and rapid synthetic process, tuneable chemical compositions and ease of modification. Among MOFs, zeolitic imidazolate framework-8 (ZIF-8) was the fondest drug carrier because of its good biocompatibility and excellent pH-responsive degradability for controlled drug release.^18,19^ Consequently, ZIF-8 was chosen as a promising drug carrier platform with successful drug encapsulation to raise chemotherapy performance. On the other hand, folate (FA)-mediated targeting has been widely used because that FA can be recognized by FA receptors overexpressed on the surfaces of the cancer cells and the receptor mediated endocytosis can increase the intracellular accumulation of drugs.^20–22^ Polyethylene glycol (PEG) was thought to be the safest material to modify nanoparticles to improve the biocompatibility and prolong the circulation time.^23^ Therefore, PEG–FA was coated on ZIF-8, which enables the better stability and tumor targeting of nanomaterials.

**Scheme 1:**
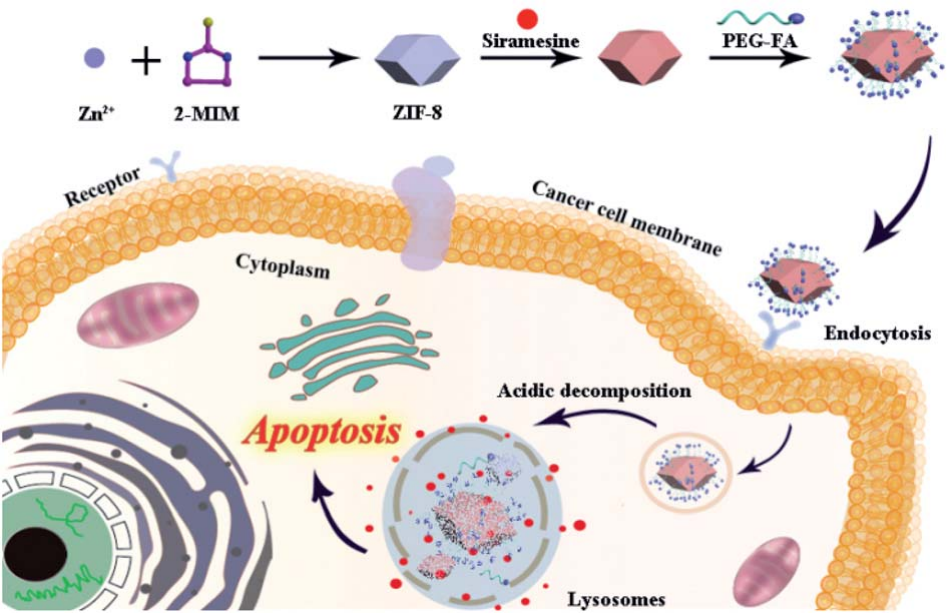
Schematic illustration of the synthetic process and therapeutic functions of the ZIF-8@Sira/FA.

Herein, we construct a new nanosystem (defined as ZIF-8@Sira/FA) based on non-anticancer drug siramesine and ZIF-8 to attain satisfactory intracellular delivery and overcome MDR. As illustrated in Scheme 1, ZIF-8 was synthesized as an ideal drug-loading nanoplatform and siramesine was then encapsulated in the ZIF-8. PEG–FA functionalized ZIF-8@Sira was obtained by coordination reaction, which can recognize the cancer cells and accelerate internalization *via* FA receptor-mediated endocytosis. After that, the pH-sensitive ZIF-8 was able to release drugs in the acidic endo/lysosomal environment and the efflux of siramesine trended to kill cancer cells by prompting LMP. More importantly, the results of antitumor activity assays *in vitro* revealed that ZIF-8@Sira/FA nanoparticles (NPs) displayed high cytotoxicity against MCF-7 and even MCF-7/ADR cells while low cytotoxicity toward normal cells. Hence, this work offers the great potential applications of non-anticancer drugs for cancer therapy and broadens the utilization of ZIF-8 in the biomedical field with higher anticancer activity and lower side effects.

## 2. Experimental section

### 2.1 Materials and reagents

2-Methylimidazole (2-MIM), zinc acetate, 5(6)-aminofluorescein were purchased from Aladdin Reagent (Shanghai, China). Siramesine hydrochloride was obtained from Shanghai Jiyi Biopharma Technology Co. Ltd. (Shanghai, China). PEG–FA and PEG–NHS were acquired from Shanghai Ziqi Biological Technology Co. Ltd. (Shanghai, China). The MCF-7 cell line was acquired from the Cell Bank of Type Culture Collection of Chinese Academy of Sciences (Shanghai, China), and the MCF-7/Adriamycin (ADR) cell line was obtained from ATCC (Shanghai, China). The Cell Counting Kit-8 (CCK-8) was purchased from Dojindo Laboratories Technologies (Kumamoto, Japan). Alexa Flour 488-dextran (10 000 MW) was purchased from Thermo Fisher Scientific Inc. (Waltham, MA, USA). Lactate dehydrogenase (LDH) assay kit, Hoechst 33342 (cellular nucleus dye) staining solution and Lyso-tracker red (lysosome dye) were acquired from Beyotime Institute of Biotechnology (Shanghai, China). Ultrapure water prepared with the Milli-Q water purification system (Merck Millipore, USA) was used throughout the whole experiment.

### 2.2 Synthesis of NPs

#### 2.2.1 Synthesis of ZIF-8, ZIF-8@Sira

The ZIF-8 was prepared based on previous report with some modification.^24^ First, 2.0 mmol 2-MIM and 0.50 mmol zinc acetate were dissolved in 2.0 mL deionized water respectively. The solution of zinc acetate was quickly added into the 2-methylimidazole solution with stirring in a round-bottomed flask. The mixed solution was standing overnight after 5 minutes continuously stirred. After that, the precipitation was collected by centrifugation (9000 rpm, 10 min) and subsequently washed with water and alcohol for more than three times to remove unreacted reagent. The product was dried overnight under vacuum at 60 °C.

To prepare drug-loaded ZIF-8, 6.0 mL of siramesine (1 mg mL^−1^) was incubated with 60 mg as-synthesized ZIF-8. After being stirred overnight in the dark, the siramesine-loaded ZIF-8 (ZIF-8@Sira) was collected by centrifugation (9000 rpm, 10 min) and washed with water and alcohol for several times to get rid of unreacted reagent. Then, ZIF-8@Sira was dispersed in deionized water for subsequent experiments.

To evaluate the drug loading efficiency, the supernatant containing unreacted siramesine was collected during the purification progress. The ultraviolet-visible (UV-vis) characteristic absorbance of siramesine was measured at 296 nm by a UV2450 spectrophotometer (Shimadzu, Japan). And the standard curve was drawn from a series of standard solutions to identify the amount of drug in the supernatant. Then the loading efficiency of siramesine was calculated as follows:
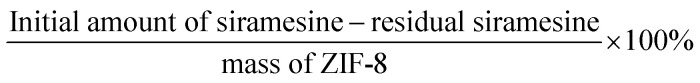


#### 2.2.2 Synthesis of ZIF-8@Sira/FA, ZIF-8/FA

To endow ZIF-8@Sira with cancer cell targeting capacity, the PEG–FA was conjugated on the surface of ZIF-8@Sira.^23^ Briefly, 20 mg PEG–FA was dispersed in as-prepared ZIF-8@Sira aqueous solution. Then, the mixture was reacted in the dark with slightly stirring for 48 h, and the precipitation was obtained by centrifugation (9000 rpm, 10 min) and subsequent several washings with ultrapure water and ethanol. All of the experiments were performed at room temperature. ZIF-8/FA was obtained *via* a similar method in the absence of siramesine. Moreover, samples for transmission electron microscope (TEM) and dynamic light scanning (DLS) were prepared by re-dispersing some product in water. Materials for other characterizations were obtained by drying at 60 °C under vacuum.

#### 2.2.3 Synthesis of ZIF-8@Sira/F

Fluorophore-labelled ZIF-8@Sira (defined as ZIF-8@Sira/F) was synthesized to track the location of the nanomaterial in the cell. First, 50 mg PEG–NHS and 30 mg 5(6)-aminofluorescein were dispersed in 5 mL H_2_O and stirred for 8 h in the dark. The solution was then transferred to the dialysis bag (MW = 1000). Owing to the elementary reactions between NHS and NH_2_, the PFG–fluorescein (PEG–F) was collected by dialysis in water for 48 h. Finally, PEG–F was dispersed in ZIF-8@Sira aqueous solution and then incubated under gentle stirring for 12 h. After washed and centrifuged three times, the ZIF-8@Sira/F was obtained and collected by centrifugation and washing.

### 2.3 Characterization

A JEM-1200EX (JEOL Technics, Tokyo, Japan) transmission electron microscope was used for TEM imaging. The hydrodynamic diameters, size distribution and zeta potential of nanoparticles were detected by a Zeta sizer Nano-ZS90 (Malvern, UK). Fourier transform infrared (FT-IR) spectra of the samples were conducted using a 5700 FT-IR spectrometer (Nicolet, USA). The X-ray diffraction (XRD) was performed on a D8 Advance Power X-ray Diffractometer (Bruker, Germany) over the 2*θ* range 5–90°.

### 2.4 *In vitro* pH-sensitive drug release study

Briefly, 1.0 mg ZIF-8@Sira was fetched accurately and put into the 2.0 mL phosphate buffer saline (PBS) with pH 7.4 and 5.0. The solution was then kept at 37 °C and gently shaken at the rate of 250 rpm. At each set time, the release medium was taken out by centrifugation and replaced with the equal volume of fresh PBS. A UV2450 spectrometer was applied to measure the absorbance of siramesine in the supernatant, which was used to calculate the drug release from the ZIF-8@Sira.

### 2.5 Cell culture

The anticancer activities of nanomaterials were determined on three types of cell lines, *i.e.*, the human normal mammary epithelial cell (MCF-10A), human breast cell line (MCF-7) and its drug resistant cell (MCF-7/ADR). Cells were incubated in Dulbecco's Modified Eagle's Medium (DMEM; MCF-7) or Roswell Park Memorial Institute (RPMI) 1640 medium (MCF-7/ADR, MCF-10A) with 10% fetal bovine serum (FBS) and 100 U mL^−1^ of penicillin and streptomycin. In addition, the cells were all kept in an INCO 108 CO_2_ Incubator (Memmert, Germany) at 37 °C with 5% CO_2_ atmosphere.

### 2.6 Anticancer activity

The cytotoxicity of ZIF-8@Sira/FA, ZIF-8@Sira and free siramesine against MCF-7, MCF-7/ADR and MCF-10A cells were evaluated by CCK-8 method. Firstly, cells were seeded in the 96-well plates at the density of 5 × 10^3^ per well. After being incubated overnight, the culture media was replaced with the fresh complete medium with ZIF-8@Sira/FA, ZIF-8@Sira or free siramesine at various siramesine concentrations (0.1–10.0 μg mL^−1^) and the cells were immediately incubated for another 24 h or 48 h. Then, the culture medium was removed and the cell viabilities were evaluated by CCK-8 assay. By measuring the absorbance of per well at 450 nm, the cell viability and IC_50_ value which was the concentration at 50% inhibition of cell viability were calculated.

### 2.7 Confocal laser scanning microscopy (CLSM)

MCF-7 cells were seeded in 35 mm glass-bottom culture dishes at a concentration of 1 × 10^4^ cells per dish, and then treated with ZIF-8@Sira/F at a siramesine concentration of 3 μg mL^−1^ for 2 h or 6 h. At the corresponding time, the cells were washed several times with PBS (pH 7.4) to remove the residual nanomaterials. After that, cells were stained with Hoechst 33342 (100 μg mL^−1^) and Lyso-tracker red (50.0 nM) for around 10 min. At last, the fluorescence images were obtained by a confocal laser scanning microscope (Leica DM6000 B, Germany). Blue, green and red luminescent emissions from Hoechst 33342, ZIF-8@Sira/F and Lyso-tracker red were excited at the wavelength of 405, 488 and 552 nm, respectively. The emission wavelengths were ranged from 450 to 500 nm for Hoechst 33342, 500 to 525 nm for ZIF-8@Sira/F and 590 to 610 nm for Lyso-tracker red. There is no interference between these three channels.

### 2.8 Visualization of LMP

In order to visualize LMP, MCF-7 cells were seeded into 35 mm glass-bottom culture dishes at a density of 8000 cell per dish. Then, cells were incubated with Alexa Fluor 488-dextran (10 000 MW, 100 μg mL^−1^) for 1 h. And the cell culture medium was replaced by different fresh drug-containing media at siramesine concentration of 3 μg mL^−1^. Pictures was taken with a fluorescence microscope (IX70, Olympus) equipped with a 100× oil immersion objective (NA = 1.30).

### 2.9 LDH release assay

For further cytotoxicity research, LDH assay kit was used to evaluate cell apoptosis. MCF-7 cells or MCF-7/ADR cells (8 × 10^3^ per well) were seeded in 96-well plates with 100 μL medium and divided into four groups with the same ZIF-8 (300 μg mL^−1^) or siramesine (3 μg mL^−1^) concentration for 24 h. After that, the LDH release agent was added and the medium supernatant solution was collected by centrifugation at 1500 rpm for 5 min. The test solution was mixed with the above supernatant solution and LDH activity value was obtained by measuring the absorbance at 490 nm by an Infinite M200 PRO microplate reader (Tecan, Austria).

### 2.10 Live/dead cell staining

For live/dead cell staining, MCF-7 cells were seeded in a 12-well dish at a concentration of 1 × 10^4^ cells per well and incubated for 24 h. Then 3 μg mL^−1^ of siramesine, ZIF-8@Sira, ZIF-8@Sira/FA and 300 μg mL^−1^ ZIF-8 were added and incubated for 2 h. The control groups were performed with PBS. The cells were digested and then stained with calcein AM and propidium iodide (PI) for another 15 min and imaged by a fluorescence microscope (IX70, Olympus).

### 2.11 Statistical analysis

Data were described as the mean ± standard deviation (SD), and statistical analysis was performed using a one-way analysis of variance (ANOVA). Statistical significance was set as *p < 0.05, and significant difference was set at **p < 0.01 and ***p < 0.001.

## 3. Results and discussion

### 3.1 Characterizations of nanoparticles

ZIF-8 has been revealed to be a promising drug nanocarrier with high drug loading due to its simple synthetic process, large surface area and favourable biocompatibility. Briefly, the carriers of the drug delivery nanosystem were first constructed by coordination between Zn^2+^ and 2-MIM. Due to the chelation with Zn^2+^ and π–π interaction with 2-MIM, siramesine was then loaded into the ZIF-8 in the second step of the synthesis process.^25–27^ PEG modification on the surface of the drug delivery nanosystem can increase its biocompatibility, while the FA can enhance its cancer cell targeting. Thus, PEG–FA was selected for the final nanoparticle modification.

**Fig. 1:**
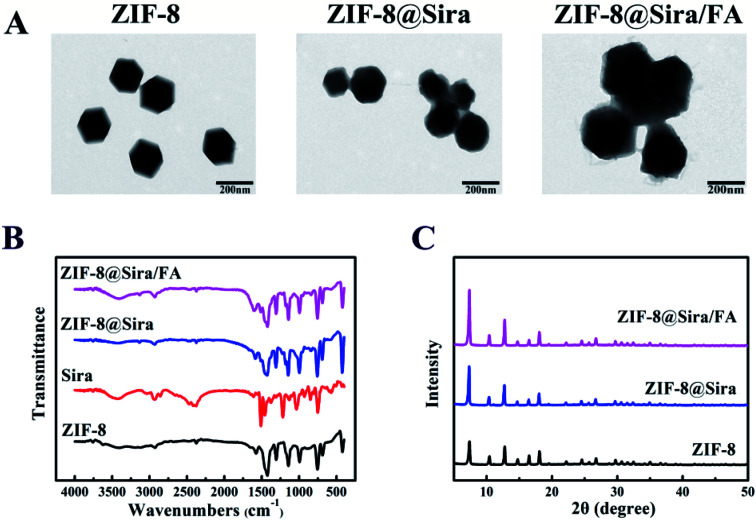
Characterization of nanoparticles. (A) TEM images of ZIF-8, ZIF-8@Sira and ZIF-8@Sira/FA, scale bar is 200 nm; (B) FTIR of ZIF-8, Sira, ZIF-8@Sira and ZIF-8@Sira/FA; (C) XRD of ZIF-8, ZIF-8@Sira and ZIF-8@Sira/FA.

After the material synthesis, TEM characterization was carried out to observe the morphology of ZIF-8, ZIF-8@Sira and ZIF-8@Sira/FA. From the TEM images in Fig. 1A, the obvious cube structure of ZIF-8 can be seen, which had sharp edges and corners. Compared with rhombic dodecahedral shape of ZIF-8, the morphology evolution to irregular shapes of ZIF-8@Sira and ZIF-8@Sira/FA signifies the guest molecules encapsulation within ZIF-8 hosts and successful surface modification. And the average diameters of the as-prepared ZIF-8, ZIF-8@Sira, ZIF-8@Sira/FA are 157 ± 5.8 nm (mean ± SD, *n* = 100), 168 ± 7.4 nm and 193 ± 6.3 nm, respectively. The dynamic light scattering (DLS) was also carried out to confirm the narrow size distribution and good dispersibility of ZIF-8 NPs (polydispersity index less than 0.5), and the hydrodynamic diameters are 188 ± 6.1 nm for ZIF-8, 209 ± 5.8 nm for ZIF-8@Sira and 281 ± 5.5 nm for ZIF-8@Sira/FA (Fig. S1 and Table S1†). It is obvious that the larger size of ZIF-8@Sira/FA is due to the coating of PEG–FA. Photographic images of ZIF-8 NPs (Fig. S2†) show the visual changes in the stepwise synthesis of the materials. And the resultant color change of ZIF-8@Sira/FA also confirms the successful modification with targeting agent.

As shown in Fig. S3,† the nitrogen adsorption/desorption analysis of ZIF-8 NPs present a type I curve accompanied by a hysteresis loop, indicating that the ZIF-8 is microporous with the surface area of 615.8 m^2^ g^−1^. With the siramesine loaded, the surface area of ZIF-8@Sira increased to 932.4 m^2^ g^−1^, which indicates that siramesine is neither on the surface nor in the inner pore of the ZIF-8, but forms a new microporous structure by combining with zinc ion. FT-IR spectra were used to evaluate the synthesis of the ZIF-8@Sira/FA (Fig. 1B). The absorption peak at 1144 cm^−1^ is the imidazole skeleton telescopic vibration from ZIF-8, while the peak at 420 cm^−1^ corresponds to the Zn–N vibration.^28,29^ After the drug loaded, the increase of peak intensity at 420 cm^−1^ and the red shift of the vibration peak at 1500 cm^−1^ of ZIF-8 indicate that the siramesine chelate with the unsaturated Zn^2+^ on the external surface of ZIF-8. Moreover, the stretches characteristic of PEG–FA at 1603 cm^−1^ and 3429 cm^−1^ are C=O in amide bond and C–H on benzene, respectively, which directs the present of PEG–FA in ZIF-8@Sira/FA.

Then, the X-ray diffraction (XRD) pattern (Fig. 1C) data confirm that ZIF-8@Sira and ZIF-8@Sira/FA maintain the same crystalline with ZIF-8, suggesting that the functional modification does not alter the crystal structure of the host material. The zeta potentials of ZIF-8, ZIF-8@Sira, ZIF-8@Sira/FA were measured to be 16.5 ± 2.4 mV, 33.8 ± 4.2 mV and −25.8 ± 1.1 mV (Fig. S4†). The stronger positive charge of ZIF-8@Sira than ZIF-8 indicates the successful loading of siramesine, which is conductive for the next electrostatic binding reaction. Compared with others, PEG–FA-coated material has a negative surface charge property, which attributed to carboxylic ions of PEG–FA, meaning the thriving electrostatic interaction between PEG–FA and ZIF-8 nanoparticles and the triumphant modification of PEG–FA on the surface of the ZIF-8 nanoparticles. All these results confirm the successful synthesis of ZIF-8@Sira/FA as expected.

### 3.2 Drug release assay *in vitro*

We evaluated the drug loading content of ZIF-8@Sira utilizing UV-vis spectrophotometry (Fig. S5A†). The calculated data gave the loading capacity of ∼94.8 μg mg^−1^ for encapsulated siramesine and the loading efficiency was 9.5%.

Then *in vitro* drug release of siramesine was investigated in PBS of different pH values. pH 7.4 representing the physiological conditions with neutral pH, while pH 5.0 condition was chosen because it represented the acidic condition of endosome in tumor cells.^30,31^ As shown in the Fig. S5B,† there is little release of siramesine (∼6%) from ZIF-8@Sira after 48 h incubation at pH 7.4. However, more than 70% of the encapsulated siramesine is released within 48 h at pH 5.0, indicating the superior pH-responsive drug release of ZIF-8 nanoparticles under the acidic endo/lysosomal environment. The long-term stability in physiological environment and the effective drug release in acidic condition of ZIF-8 NPs illustrate a favourable drug delivery system for the cancer therapy.

To calculate the number of PEG on each ZIF-8 particle, the single molecule fluorescence imaging (Fig. S6†) of PEG–F and ZIF-8@Sira/F were obtained by a CLSM (Leica TCS SPE, Germany) with a 488 nm laser excitation source. Through the comparative analysis of the fluorescence intensity of the material by Image-J, the mean fluorescence intensity (MFI) of PEG–F is 0.09, and that of ZIF-8@Sira/F is 0.25 as shown in Table S2.† The loading capacity of PEG–FA was calculated about 3 PEG molecules loaded on one ZIF-8 particles.

### 3.3 Antitumor activity of drug delivery systems

**Fig. 2:**
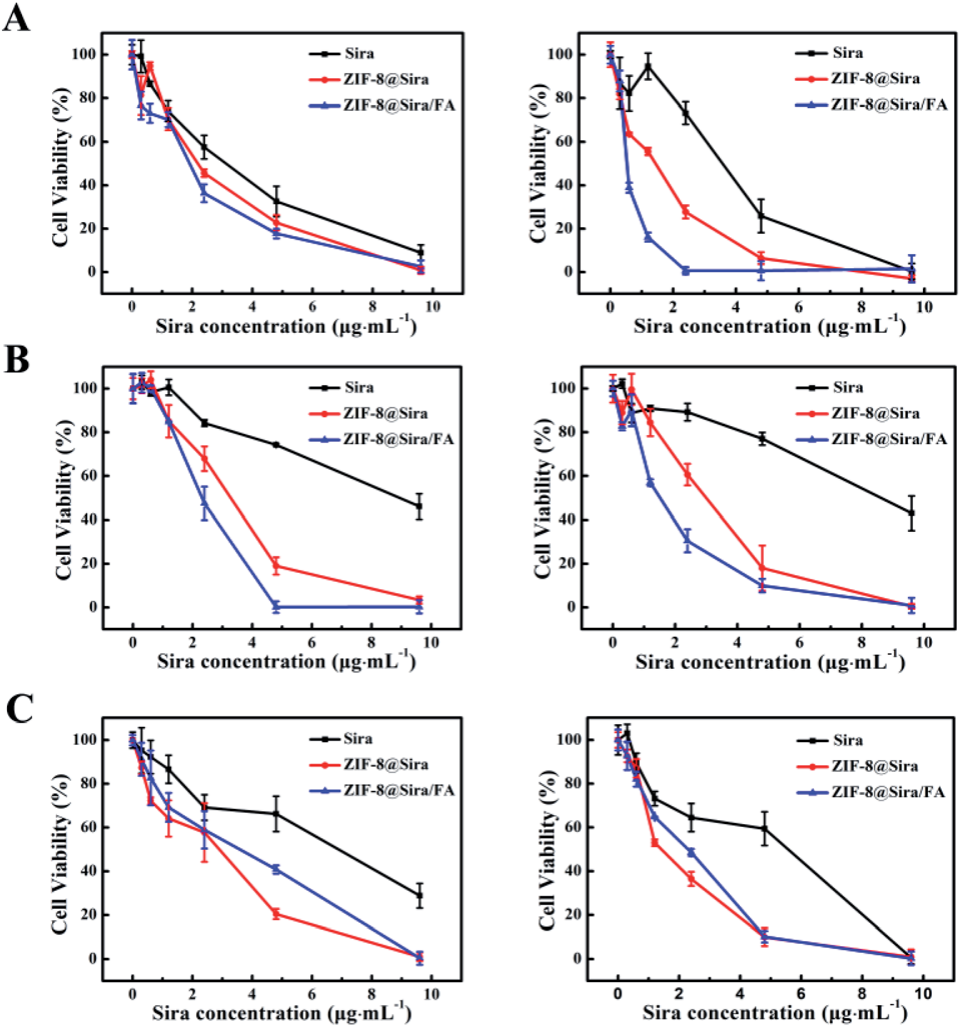
Antitumor activity of drug delivery system. Cell viability of MCF-7 cells (A), MCF-7/ADR cells (B) and MCF-7/10A cells (C) after incubating with free Sira, ZIF-8@Sira or ZIF-8@Sira/FA at different concentrations for 24 h (left) and 48 h (right), the data are represent as the mean ± SD (*n* = 6).

Encouraged by the efficient drug release of ZIF-8@Sira under acidic endosomal environment, we further quantitatively investigated cytotoxicity of drug delivery systems on three kinds of cells. Firstly, we confirmed that non-anticancer drug siramesine did have anticancer activity just as reported in the previous work^6^ by incubating a series of concentrations (0.3 to 9.6 μg mL^−1^) of siramesine with MCF-7 cells for 24 or 48 h, and the half-maximum inhibitory concentration values (IC_50_) are 2.72 and 3.32 μg mL^−1^, respectively (Table S3, ESI†). Compared with other traditional chemotherapy drugs,^32–34^ the MCF-7 cells is susceptible to siramesine. In order to explore the cytotoxicity of the drug delivery nanosystem constructed in this work, ZIF-8@Sira and ZIF-8@Sira/FA were also discussed for comparison. As we expected, the inhibition rates of ZIF-8@Sira and ZIF-8@Sira/FA are higher than that of free siramesine to MCF-7 cells (Fig. 2A and Table S3†). It is mainly because that the free drugs are internalized by passive diffusion, while the drug delivery nanosystem can be taken in cells *via* endocytosis, resulting in higher cellular uptake rate and enhanced cellular accumulation.^20,35^ Hence, ZIF-8 based drug delivery system is of great advantage. It is worth noting that ZIF-8@Sira/FA has a higher rate of cell inhibition than ZIF-8@Sira, which is obviously caused by effective combination of the FA in the ZIF-8@Sira/FA and the FA receptors overexpressed on the surfaces of the cancer cells. It shows the excellent cancer cell targeting of the materials in our work.

MDR is the primary obstacle to cancer overcoming.^13^ To study the ability of drug delivery system for MDR surmounting, MCF-7/ADR cells were incubated with different materials. Interestingly, we found that free siramesine at a low concentration (0 to 9.6 μg mL^−1^) did not exhibit visible cytotoxicity on MCF-7/ADR cells (Fig. 2B). However, after loaded on the ZIF-8, siramesine kills the MCF-7/ADR cells more effectively. The IC_50_ values are 8.57, 2.07 and 1.46 μg mL^−1^ of free siramesine, ZIF-8@Sira and ZIF-8@Sira/FA, respectively (Table S3†). A 5.9-fold reduction in the above IC_50_ values after 48 h incubation with MCF-7/ADR cells means the effective solution of the multidrug resistance of cancer cells. Siramesine acts directly on lysosomes and induces apoptosis through lysosomal permeability. When the siramesine enters the cell, it is greatly diluted in the cytoplasm before reaching the action site (lysosome). Thus, nanoscale host materials can directly enter the lysosome, which can overcome the dilution of drugs and reduce the potential side effects. And ZIF-8@Sira/FA can promote intracellular uptake through endocytic pathways, which is beneficial for drugs to avoid the efflux associated with MDR.^34^

A good drug delivery nanosystem should have low toxicity to normal cells in addition to excellent antitumor activity. Therefore, we investigated the impact of nanoparticles on MCF-10A and the results shown in Fig. 2C. Compared with ZIF-8@Sira, ZIF-8@Sira/FA exhibit decreased cytotoxicity against MCF-10A. The PEG–FA modification can reduce the damage to normal cells because of the specific identification of cancer cells by overexpressed FA receptors on the cancer cells surfaces, revealing that the ZIF-8@Sira/FA is an excellent drug delivery nanosystem with minor side effects.

Finally, we also evaluated the toxicity of ZIF-8/FA against cancer cells (Fig. S7†). After incubated 24 or 48 h, the cell viability is still above 80% even at a high concentration (100 μg mL^−1^) of ZIF-8/FA, which means the good biocompatibility and the low toxic side effects of ZIF-8. The cell death was also evaluated by live/dead cells staining (calcein AM and propidium iodide). As shown in Fig. S8,† there is no obvious cytotoxicity in both siramesine and ZIF-8 treatment groups, while the remarkable dead staining is observed in the ZIF-8@Sira/FA administration group. These results completely agree with the cell activity assay, verifying the above conclusions and excluding the toxic effects of ZIF-8.

### 3.4 Mechanism investigation

**Fig. 3:**
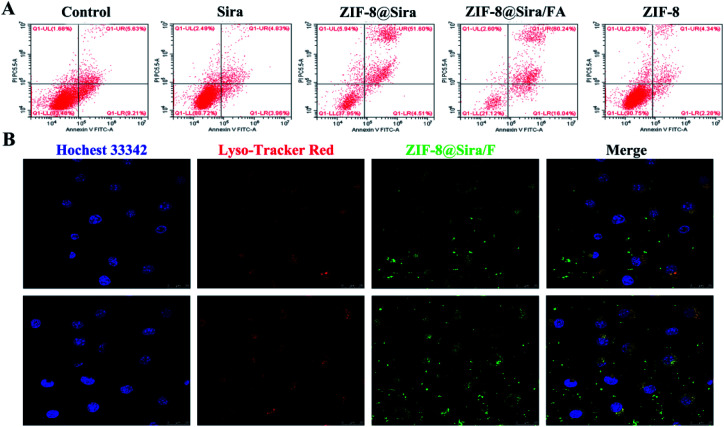
Mechanism investigation. (A) Flow cytogram representing apoptosis assay based on Annexin V-FITC and propidium iodide staining of MCF-7 cells after treatment with different therapeutic groups for 2 h; (B) CLSM images of the intracellular accumulation of ZIF-8/F in MCF-7 cells after incubating for 2 h (top) and 6 h (bottom).

As mentioned above, ZIF-8@Sira/FA can effectively release drugs in acid environment and have strong antitumor activity with slight cytotoxicity to normal cells. The mechanism of cell death caused by ZIF-8@Sira/FA has been further explored by flow cytometry. As presented in Fig. 3A, free siramesine is slight toxic to MCF-7 cells at a low concentration of 3 μg mL^−1^, thus there is no difference between the free drug group and the control group (cell culture medium without drugs). Similar result of the ZIF-8 group *versus* the free drug group indicates the good biosafety of ZIF-8. In addition, the proportions of apoptosis cells are 56.11% and 76.28% after treatment with ZIF-8@Sira and ZIF-8@Sira/FA (at a siramesine concentration of 3 μg mL^−1^), which significantly induce apoptosis especially late apoptosis of cells after 2 h incubation. The results suggest us that the nanomaterials can be rapidly enriched in cells and causes rapid induction of cell death.

Based on the above experiments, we further confirmed the material internalization and anticancer mechanism. Internalization of drug delivery system into cells plays an important role in effective anticancer treatment. Since neither the ZIF-8 nor the siramesine is fluorescent, we first connected 5(6)-aminofluorescein on the surface of the ZIF-8@Sira for fluorescent labelling. Then the cellular uptake of ZIF-8@Sira/F on MCF-7 cells was evaluated by CLSM. From the fluorescence images in Fig. 3B, we can see the co-localization of ZIF-8@Sira/F with lysosomes in the cytosol after 2 h incubation. With the time prolonging, more and more fluorescent dyes are observed in the cytosol and even the nuclei, indicating the drug distribution after the collapse of the material structure. Overall, the results suggest that drug delivery system enter into lysosomes quickly and degrade in the endo/lysosome acid condition. Subsequently, the drug release will lead to effective endo/lysosome escape and further cause the cell death.

**Fig. 4:**
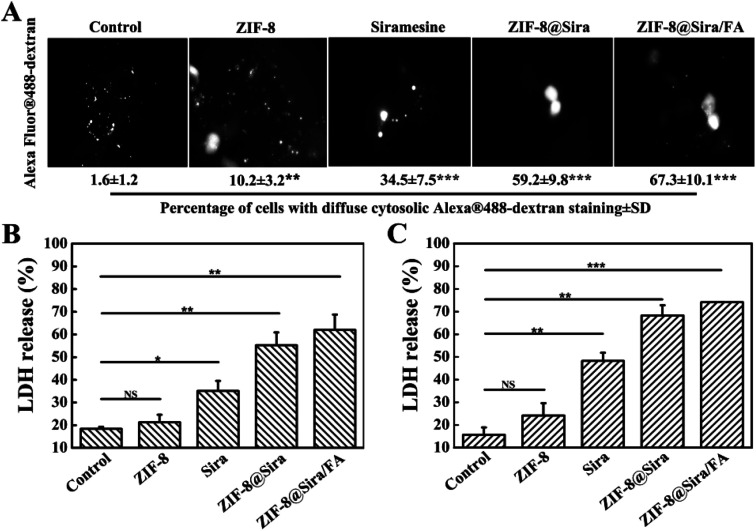
Mechanism investigation. (A) Visualization of LMP, ***p < 0.001 when compared drug treated cells with untreated cells (control); LDH assay of (B) MCF-7 cells and (C) MCF-7/ADR cells after incubated with different materials.

ZIF-8 has been repeatedly proven to be a biosafety nanocarrier, so the anticancer activity of ZIF-8@Sira/FA is due to the effective internalization and release of the siramesine. The previous work has reported that siramesine can induce LMP, further triggering the leakage of lysosomal proteases to the cytosol. Alexa Fluor 488-dextran was used to detect the integrity of the lysosomal membranes.^36^ After the lysosomal membrane permeability increased, fluorescence will change from a punctate lysosomal to diffuse cytosolic staining. As shown in Fig. 4A, free siramesine increase LMP slightly, whereas ZIF-8@Sira and ZIF-8@Sira/FA dramatically enhanced LMP. ZIF-8@Sira/FA has a higher diffuse staining than ZIF-8@Sira owing to the more drug accumulation of ZIF-8@Sira/FA through FA-mediated endocytosis. The released drug in the cancer cells can induce LMP and then cause apoptosis which may cause the cell membrane rupture. The destruction of cell membrane structure caused by apoptosis can lead to the release of LDH in the cytoplasm into the culture medium. Thus, analysis of cell death also can be achieved by detecting the activity of LDH. The LDH release assay on MCF-7 (Fig. 4B) and MCF-7/ADR (Fig. 4C) cells also indicate the overt toxicity of ZIF-8@Sira/FA, slight toxicity of siramesine and the negligible toxicity of ZIF-8. And their consistent trend means that the drug delivery nanosystem constructed in our work kill the cancer cells from the inside by the lysosomal cell death pathway, overcoming the MDR.

In summary, we can find the mechanism of ZIF-8@Sira/FA for the selective killing of cancer cells: the drug delivery nanosystems first enter the lysosome by endocytosis, and decompose in the acidic environment of lysosome to release the drug, causing LMP and subsequent release of LDH, resulting in the apoptosis of cells. In the last, we further verified the FA and FA receptor mediated endocytosis through competition experiment. As shown in Fig. S9,† free FA (5 μg mL^−1^) has negligible toxicity to cancer cells, while ZIF-8@Sira/FA has obvious toxicity to cancer cells. However, in the presence of 5 μg mL^−1^ free FA, the antitumor activity of ZIF-8@Sira/FA decreased. This result also illustrates the excellent targeting of the drug delivery system in our work. FA modification of the ZIF-8@Sira/FA reduces the side effects on normal tissue and increases the accumulation of materials in the cancer cells, providing the basis for controlled drug release and outstanding cancer inhibition of the materials.

## 4. Conclusion

In this work, we construct a new drug delivery nanosystem based on non-anticancer drug siramesine and ZIF-8 for anticancer therapy. ZIF-8@Sira/FA was successfully synthesized owing to the chelation of siramesine and Zn^2+^ and the electrostatic interaction between ZIF-8@Sira and PEG–FA. Due to the ideal size and FA modification, the ZIF-8@Sira/FA can accumulate quickly in cancer cells and then release drugs in the acidic endo/lysosomal environment. Subsequently, the efflux of siramesine trends to trigger apoptosis by prompting LMP. *In vitro* cytotoxicity tests on MCF-7 and MCF-7/ADR cells attain satisfactory anticancer effect and negligible side effects. Thus, this work provides a potential application of non-anticancer drugs for cancer therapy through lysosomal cell death pathway and expands the utilization of ZIF-8 in the biomedical field for anticancer therapy.

## Conflicts of interest

The author(s) declare that they have no competing interests.

## Supplementary Material

RA-010-C9RA09923A-s001
