# Supplementary material for: A siramesine-loaded metal organic framework nanoplatform for overcoming multidrug resistance with efficient cancer cell targeting
Source: RSC Adv. 2020 Feb 14;10(12):6919–26. doi: 10.1039/c9ra09923a (PMC9049735; doi:10.1039/c9ra09923a)
Supplement: RA-010-C9RA09923A-s001 [file RA-010-C9RA09923A-s001.pdf]

Electronic Supplementary Material (ESI) for RSC Advances.
This journal is © The Royal Society of Chemistry 2020

Electronic supplementary information (ESI)

Siramesine-loaded Metal Organic Framework Nanoplatform for Overcoming Multidrug Resistance with Efficient Cancer Cell Targeting

Jiahui Liu [1], Menghuan Tang [1], Yanghao Zhou [2], Yijuan Long [1], Yuan Cheng [2], Huzhi Zheng [1*]

[1] Key Laboratory of Luminescent and Real-Time Analytical Chemistry (Southwest University), Ministry of Education, College of Chemistry and Chemical Engineering, Southwest University, Chongqing, 400715, P. R. China.

[2] Department of Neurosurgery, the Second Affiliated Hospital of Chongqing Medical University, Chongqing, 400010, P. R. China

*Corresponding author; E-mail: zhenghz@swu.edu.cn (H. Zheng).

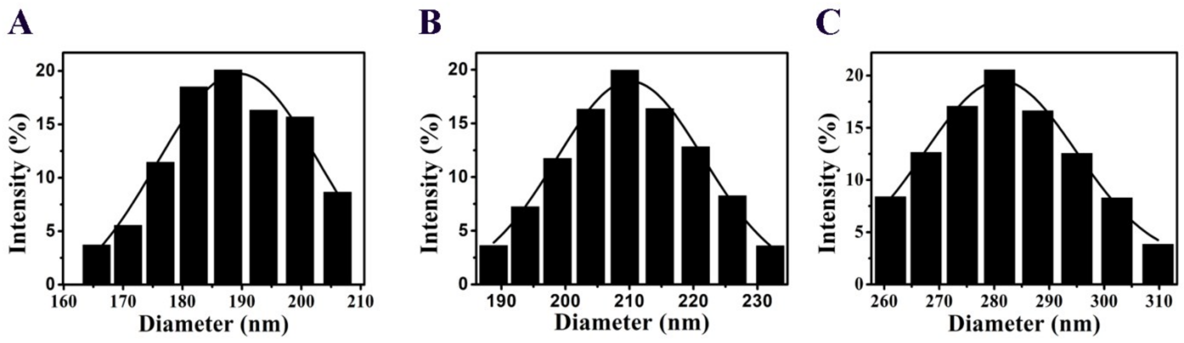

Figure S1. DLS analysis of ZIF-8, ZIF-8@Sira and ZIF-8@Sira/FA.

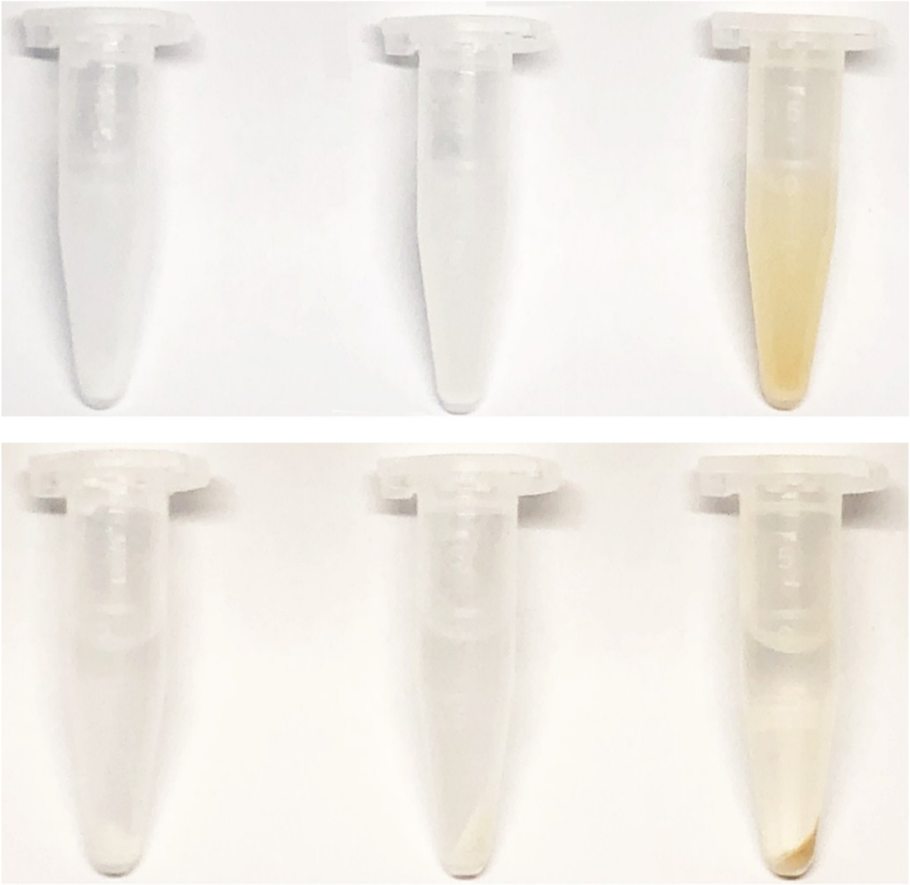

ZIF-8 **ZIF-8@Sira** **ZIF-8@Sira/FA**

Figure S2. Photographic images of ZIF-8, ZIF-8@Sira and ZIF-8@Sira/FA aqueous solution before (top) and after (bottom) centrifugation.

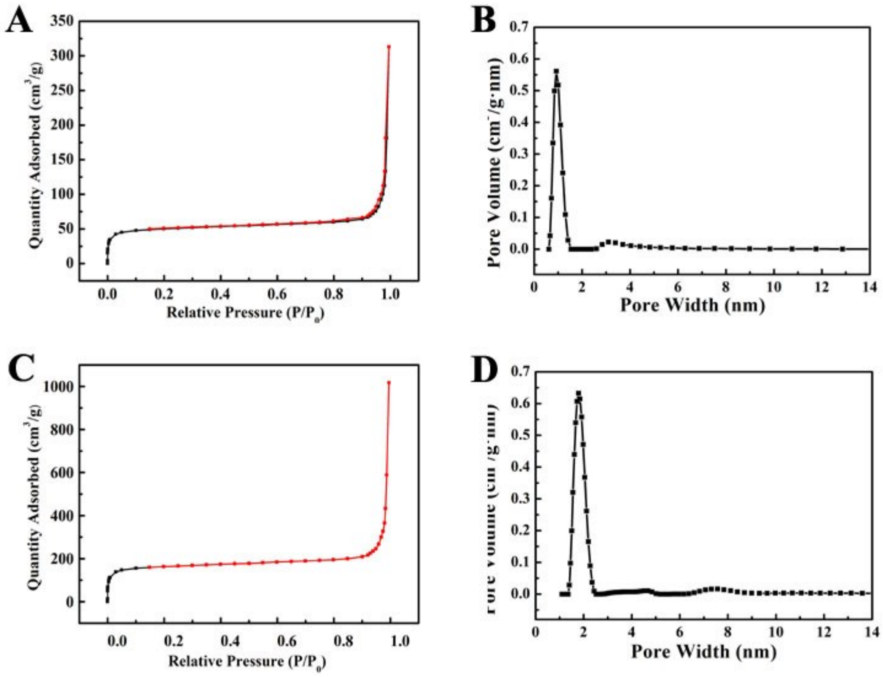

Figure S3. Nirogen adsorption-desorption isotherms of ZIF-8 (A) and ZIF-8@Sira (C) and the pore size distribution of ZIF-8 (B) and ZIF-8@Sira (D).

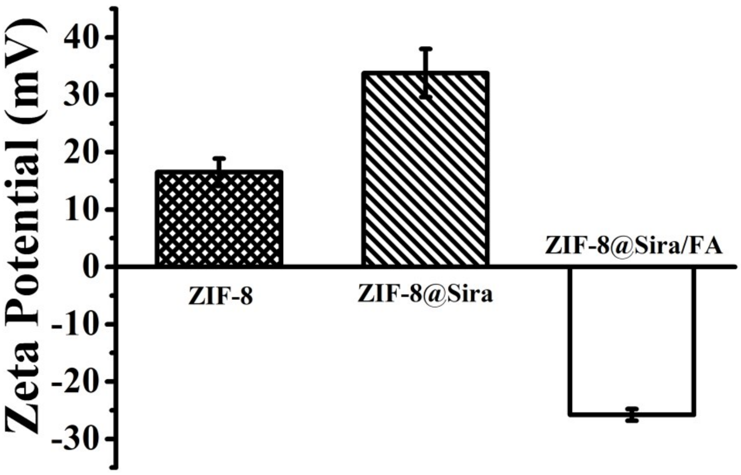

Figure S4. Zeta potentials of ZIF-8, ZIF-8@Sira and ZIF-8@Sira/FA, bars represent mean ± standard deviation (n = 3).

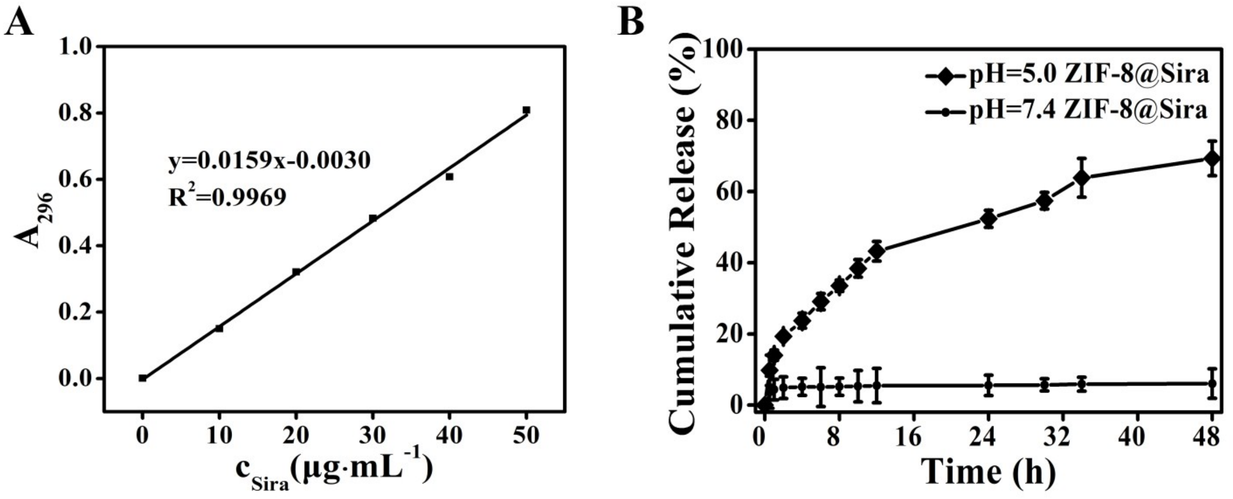

Figure S5. Drug release assay in vitro. (A) Linear calibration plots for siramesine detection; (B) The release curves of siramesine from ZIF-8@Sira in different PBS of pH 7.4 and pH 5.0 at 37 ℃.

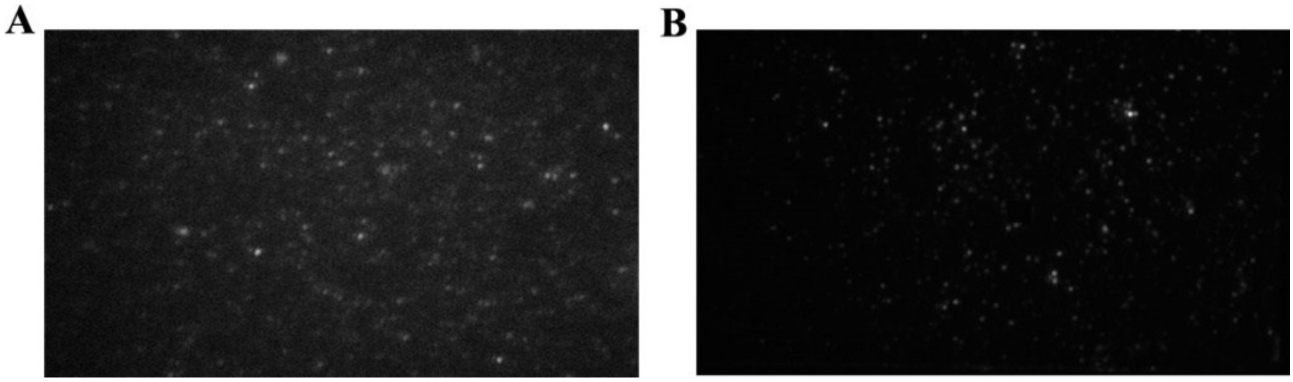

Figure S6. Single molecule fluorescence imaging of PFG-F (A) and ZIF-8/F (B).

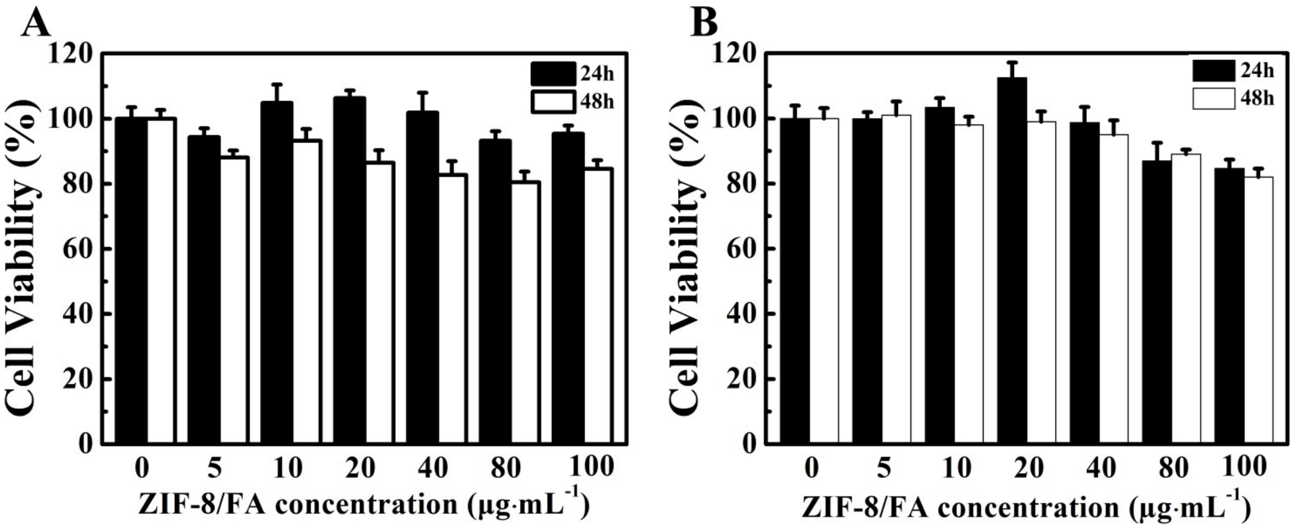

Figure S7. Cell viability of MCF-7 cells (A) and MCF-7/ADR cells (B) after incubating with ZIF-8/FA at different concentrations for 24 h and 48 h, the data are represent as the mean ± SD (n = 6).

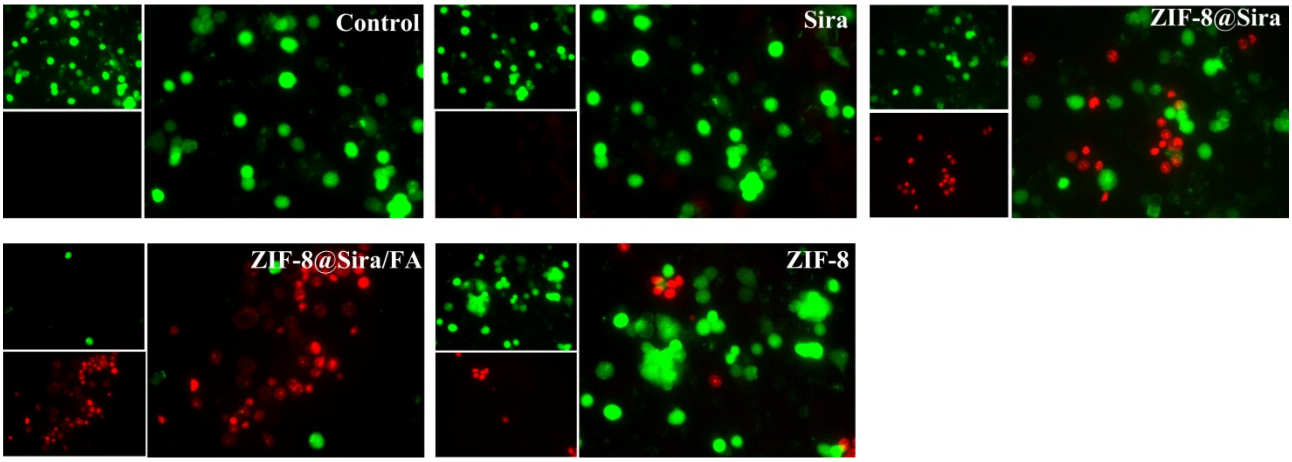

Figure S8. Confocal fluorescence images of drug delivery system incubated MCF-7 cells for 2 h. Live/dead stain shows dead cells as red and viable cells as green.

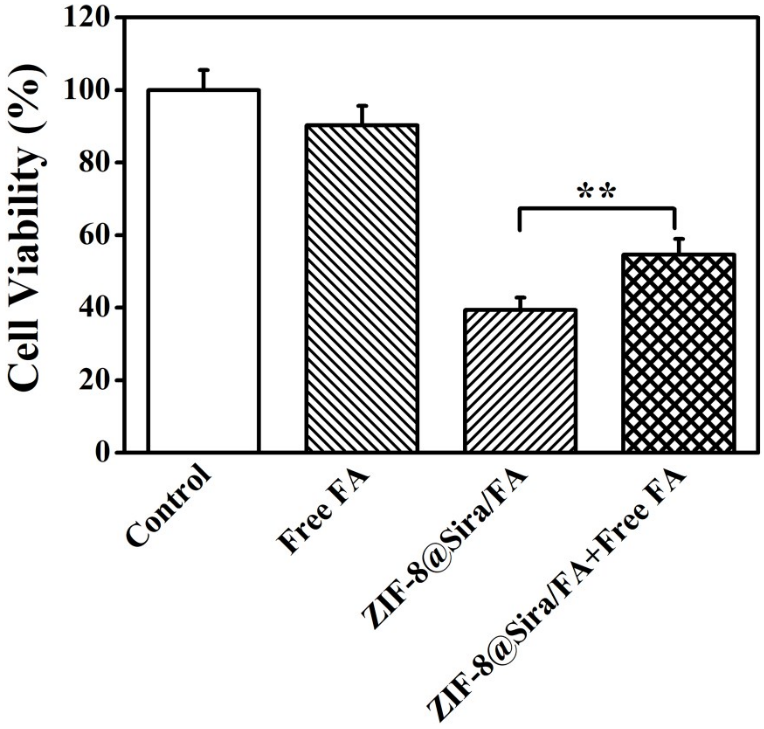

Figure S9. Cytotoxicity of ZIF-8@Sira/FA (2 μg·mL^{-1}) against MCF-7/ADR cells with free FA (5 μg·mL^{-1}) in 24 h. **p < 0.01 when compared with ZIF-8@Sira/FA group. Data are represented as mean ± SD (n = 3).

Table S1. The data obtained from TEM and DLS of NPs.

	**Diameter (nm)**	**DLS size (nm)**	**PDI**
**ZIF-8**	157 ± 6.8 (n = 100)	188 ± 6.1 (n = 3)	0.37
**ZIF-8@Sira**	168 ± 7.4 (n = 100)	209 ± 5.8 (n = 3)	0.42
**ZIF-8@Sira/FA**	193 ± 6.3 (n = 100)	281 ± 5.5 (n = 3)	0.26

Table S2. Analysis data of single molecule fluorescence imaging by Image-J.

	**Fluorescence intensity**	**Particle number**	**MFI**
**PEG-F**	207.16	2360	0.09
**ZIF-8@Sira/F**	215.84	862	0.25

Table S3. IC_{50} values for MCF-7 cells, MCF-7/ADR cells, and MCF-7/10A cells treated with ZIF-8 nanoparticles for 24 h and 48 h (n=3).

IC_{50} (μg·mL^{-1}) — 24 h	MCF-7	MCF-7/ADR	MCF-10A
**Sira**	2.72	8.64	7.46
**ZIF-8@Sira**	2.10	2.95	1.92
**ZIF-8@Sira/FA**	1.56	2.27	2.60

IC_{50} (μg·mL^{-1}) — 48 h	MCF-7	MCF-7/ADR	MCF-10A
**Sira**	3.32	8.57	5.97
**ZIF-8@Sira**	1.09	2.70	1.24
**ZIF-8@Sira/FA**	0.54	1.46	2.28
